# CRISPR/Cas9-mediated knock-in of alligator cathelicidin gene in a non-coding region of channel catfish genome

**DOI:** 10.1038/s41598-020-79409-5

**Published:** 2020-12-17

**Authors:** Rhoda Mae C. Simora, De Xing, Max R. Bangs, Wenwen Wang, Xiaoli Ma, Baofeng Su, Mohd G. Q. Khan, Zhenkui Qin, Cuiyu Lu, Veronica Alston, Darshika Hettiarachchi, Andrew Johnson, Shangjia Li, Michael Coogan, Jeremy Gurbatow, Jeffery S. Terhune, Xu Wang, Rex A. Dunham

**Affiliations:** 1 School of Fisheries, Aquaculture and Aquatic Sciences, Auburn University, Auburn, AL 36849 USA; 2 Ministry of Education Key Laboratory of Marine Genetics and Breeding, College of Marine Life Sciences, Ocean University of China, Qingdao, 266003 China; 3 Department of Pathobiology, Auburn University, Auburn, AL 36849 USA; 4 HudsonAlpha Institute for Biotechnology, Huntsville, AL 35806 USA; 5 Present Address: College of Fisheries and Ocean Sciences, University of the Philippines Visayas, 5023 Miagao, Iloilo Philippines; 6 Present Address: Department of Biological Science, Florida State University, Tallahassee, FL 32304 USA; 7 Present Address: Department of Fisheries Biology and Genetics, Bangladesh Agricultural University, Mymensingh, 2202 Bangladesh

**Keywords:** Biotechnology, Genetics, Molecular biology

## Abstract

CRISPR/Cas9-based gene knockout in animal cells, particularly in teleosts, has proven to be very efficient with regards to mutation rates, but the precise insertion of exogenous DNA or gene knock-in via the homology-directed repair (HDR) pathway has seldom been achieved outside of the model organisms. Here, we succeeded in integrating with high efficiency an exogenous alligator cathelicidin gene into a targeted non-coding region of channel catfish (*Ictalurus punctatus*) chromosome 1 using two different donor templates (synthesized linear dsDNA and cloned plasmid DNA constructs). We also tested two different promoters for driving the gene, zebrafish ubiquitin promoter and common carp β-actin promoter, harboring a 250-bp homologous region flanking both sides of the genomic target locus. Integration rates were found higher in dead fry than in live fingerlings, indicating either off-target effects or pleiotropic effects. Furthermore, low levels of mosaicism were detected in the tissues of P_1_ individuals harboring the transgene, and high transgene expression was observed in the blood of some P_1_ fish. This can be an indication of the localization of cathelicidin in neutrophils and macrophage granules as also observed in most antimicrobial peptides. This study marks the first use of CRISPR/Cas9 HDR for gene integration in channel catfish and may contribute to the generation of a more efficient system for precise gene integration in catfish and other aquaculture species, and the development of gene-edited, disease-resistant fish.

## Introduction

A powerful genome-editing tool known as clustered regularly interspaced short palindromic repeats (CRISPR)/CRISPR-associated protein 9 (Cas9) technology has been of growing use for gene editing in aquaculture^1–3^. Interest in genomic alteration of farmed fish has increased, especially after the first genetically modified fish (AquAdvantage salmon) has been approved for consumption^4^. In the CRISPR/Cas9 system, the co-delivery of endonuclease Cas9 combined with a synthetic single guide RNA (sgRNA) targeting certain gene(s) into eukaryotic cells can edit the genome by stimulating a double-strand break (DSB) at a desired site(s), and the subsequent DNA repair process could introduce indel(s)^5^. In the last five years, it has been successfully performed in several aquaculture species, including Atlantic salmon^6,7^, Nile tilapia^8^, common carp^1^, channel catfish^2,9^, sea bream^3^ and rainbow trout^10^ to generate a variety of phenotypes related to reproduction, fertility, muscle growth and disease resistance. However, the use of CRISPR/Cas9 has been largely limited to gene knock-outs in most fish species^1–3,9,10^, but has great potential for gene knock-in through homology-directed repair (HDR). The HDR pathway can allow precise integration of any desirable DNA sequence at the target site^11,12^, thus allowing the creation of gene-edited fish with desirable performance traits.

Protein coding sequences are ready targets for many CRISPR/Cas9 applications, wherein investigators have generated small insertion and deletion (indel) mutations to disrupt the open reading frames of protein coding genes^13,14^. On the contrary, mutation of non-coding sequences using CRISPR/Cas9 system is often difficult to achieve, because small indels caused by a single mutation may not result in a detectable loss of function^8^. Targeting non-coding sequences either through loss-of-function or gain-of-function approaches can be advantageous since these regions are proposed to affect the expression of neighboring or distant genes by acting as signaling, guiding, sequestering or scaffolding molecules^15,16^. Targeting non-coding sequences might also affect multiple genes resulting in off-target effects. Some studies have successfully obtained large genomic deletion using dual guide RNAs (gRNAs) in mammalian cells and animal models such as mouse and zebrafish^17,18^. Deletion mutations of up to 900 bp were recently achieved in channel catfish targeting the toll/interleukin 1 receptor domain-containing adapter molecule (TICAM 1) using a single guide RNA^9^. Moreover, Li et al.^8^ reported that the CRISPR/Cas9 system could effectively generate desirable non-coding sequence mutants in Nile tilapia (*Oreochromis niloticus*). There are non-coding regions away from genes, miRNA loci, lncRNA and heterochromatin regions, and to date, there have been no published studies of targeted gene insertion in such a genomic region. Our hypothesis is that the insertion of transgenes in such regions would decrease the possibility of knocking out valuable loci and affecting the expression of other genes. Additionally, targeting such areas for gene insertion could prevent or decrease the probability of negative pleiotropic or off-target effects.

Channel catfish (*Ictalurus punctatus*) is an important food fish in the United States that has been impacted by diseases, especially in commercial aquaculture. Efforts have been geared towards increasing disease resistance in this species as 40% of catfish production was lost to disease at the beginning of the current decade^19^ and continues to be problematic^20^. This is the case for aquaculture in general^19,21^. Improved catfish production systems have been adopted and hybrid catfish (*I. punctatus ♀* × blue catfish, *I. furcatus ♂*), which is more resistant to diseases than channel catfish, are predominantly cultured. The use of hybrids has resulted in increased farm productivity and some increase in disease resistance, but the hybrids are not totally resistant to diseases and to date, there have been few dramatic advancements in disease control^22^. One alternative strategy would be developing disease-resistant fish lines through transgenesis. However, in the past, there has been some inefficiency in the generation of transgenic lines, and targeted gene insertion was almost impossible.

One particular class of antimicrobial peptides (AMPs) that could be useful for this cause are cathelicidins, which have been shown to exhibit broad-spectrum antimicrobial activity in vitro and in vivo^23,24^. Expression of genes encoding peptides with in vitro antimicrobial activity can result in enhanced resistance to bacterial pathogens in transgenic fish^25,26^. Recently, cathelicidins derived from American alligator (*Alligator mississippiensis*) were shown to have a strong activity against some Gram-negative bacteria as well as in multi-drug resistant bacterial pathogens such as *Acinetobacter baumannii* and *Klebsiella pneumoniae*^27,28^. Cathelicidins from other species were not fully characterized and have had limited in vitro testing^23,29^. Inserting alligator cathelicidin gene in channel catfish using CRISPR/Cas9 system might enhance its resistance to various pathogens. A transgenic fish encoding an antimicrobial peptide can confer immunity in fish since it will be protected by the expressed peptide from early in development. Also, an innately disease-resistant fish would not require specific vaccination for certain pathogens, and thus, will provide an economical solution to bacterial disease problems^25^.

CRISPR/Cas9 has been explored as a knock-in system in model fishes targeting reporter/driver genes in medaka^30,31^ and zebrafish^32,33^. But limited studies for CRISPR/Cas9 knock-in were found on farmed fish^7,34^, and these studies primarily focused on non-performance genes. Here, we attempted to use CRISPR/Cas9-mediated HDR to knock-in an alligator cathelicidin gene driven by two different fish promoters and testing of two different types of donor DNA templates. Our objective was to insert this disease-resistance gene in a non-coding region of the channel catfish genome where the insertion would, theoretically not disable any important locus or function of a normal catfish. Additionally, targeting a non-coding region might allow transgene expression without suppression from neighboring genes. We aim to produce disease-resistant lines of channel catfish carrying actively expressing cathelicidin genes with positive biological functions that can be inherited by subsequent generations.

## Results

### Efficient HDR led to high integration rates

**Figure 1:**
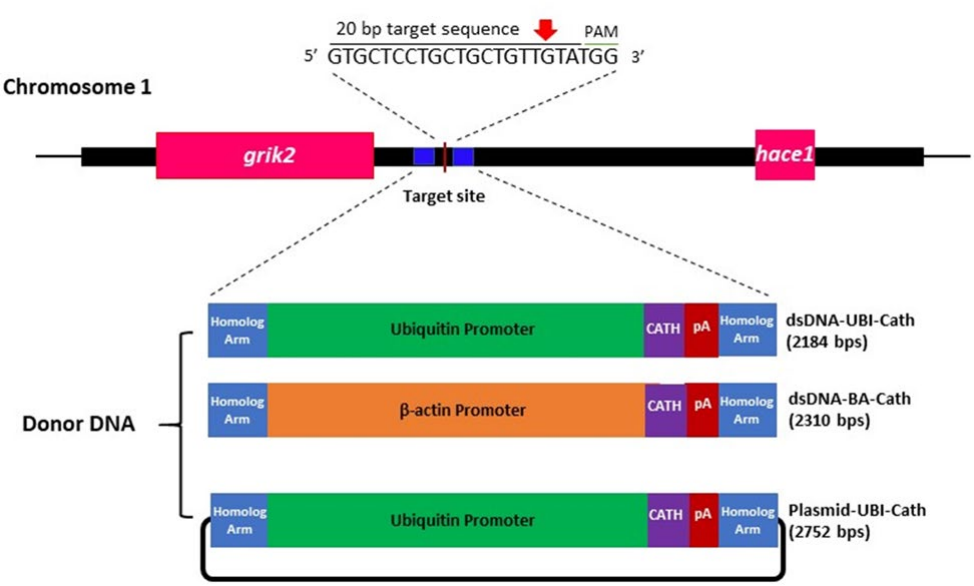
Schematic representation of the target site and the surrounding genome in chromosome 1 of channel catfish (*Ictalurus punctatus*) genome where insertion of the transgene was made. The 20-bp guide RNA sequence containing the PAM was shown and the cut site (red arrow) which aided the targeted insertion of the donor DNA constructs: double-stranded DNA (dsDNA) driven by zebrafish ubiquitin promoter (dsDNA-UBI-Cath), dsDNA driven by carp β-actin promoter (dsDNA-BA-Cath) and plasmid DNA, pUCIDT with zebrafish ubiquitin promoter (plasmid-UBI-Cath). *Grik2* is 1376 bp upstream of the target and *hace1* is 295,702 bp downstream of the target.

We developed three transgene constructs carrying the cathelicidin gene: two double-stranded DNA (dsDNA) constructs each carrying different promoters namely zebrafish (*Danio rerio*) ubiquitin promoter and common carp (*Cyprinus carpio*) β-actin promoter and a plasmid DNA construct driven by zebrafish ubiquitin promoter. Homologous arms (250 bp each), derived from chromosome 1 of channel catfish, were placed at both ends of the transgene constructs. Details on the design of the donor constructs are available in the Methods section. To insert the donor template carrying the cathelicidin gene, we used the small guide RNA (sgRNA) (Table 1) that directed Cas9 nuclease to that specific location in a non-coding region of chromosome 1 (Fig. 1). The integration site lies in a 297,098 bp non-coding window with no genes, microRNAs, long non-coding RNAs and heterochromatin binding sites, with the exception of two long non-coding RNAs (coordinates 19,220,890–19,222,416 and 19,318,070–19,319,829) which were avoided as to prevent knocking out any important functions.

**Table 1:** The sequences of small guide RNA (sgRNA) and the universal (common) primer used to target chromosome 1 (Chr1) of channel catfish (*Ictalurus punctatus*).

Guide RNA	Oligo sequence (5′–3′)
Chr1 sgRNA	GTGCTCCTGCTGCTGTTGTATGG
Universal primer	TTTTGCACCGACTCGGTGCCACTTTTTCAAGTTGATAACGGACTAGCCTTATTTTAACTTGCTATTTCTAGCTCTAAAAC

Underlined sequences represent the protospacer adjacent motif (PAM).

Successful integration of all three constructs was detected in both dead and living fry. Integration was confirmed to have occurred at the targeted site on chromosome 1 of channel catfish (Database ID: NC_030416.1; coordinate 19,129,218). While no genes are encoded in this genomic window, the areas upstream and downstream are gene rich (seven genes within 500,000 bps upstream and six genes within 500,000 bps downstream), increasing the probability of transgene expression. The closest genes to the integration site are *grik2* (glutamate receptor iontropic kainate 2; coordinates 18,964,226–19,127,825) upstream and *hace1* (HECT domain and ankyrin repeat containing E3 ubiquitin protein; coordinates 19,424,923–19,448,550) downstream, the latter of which contains a ubiquitin promoter similar to two of transgene constructs (Fig. 1).

**Figure 2:**
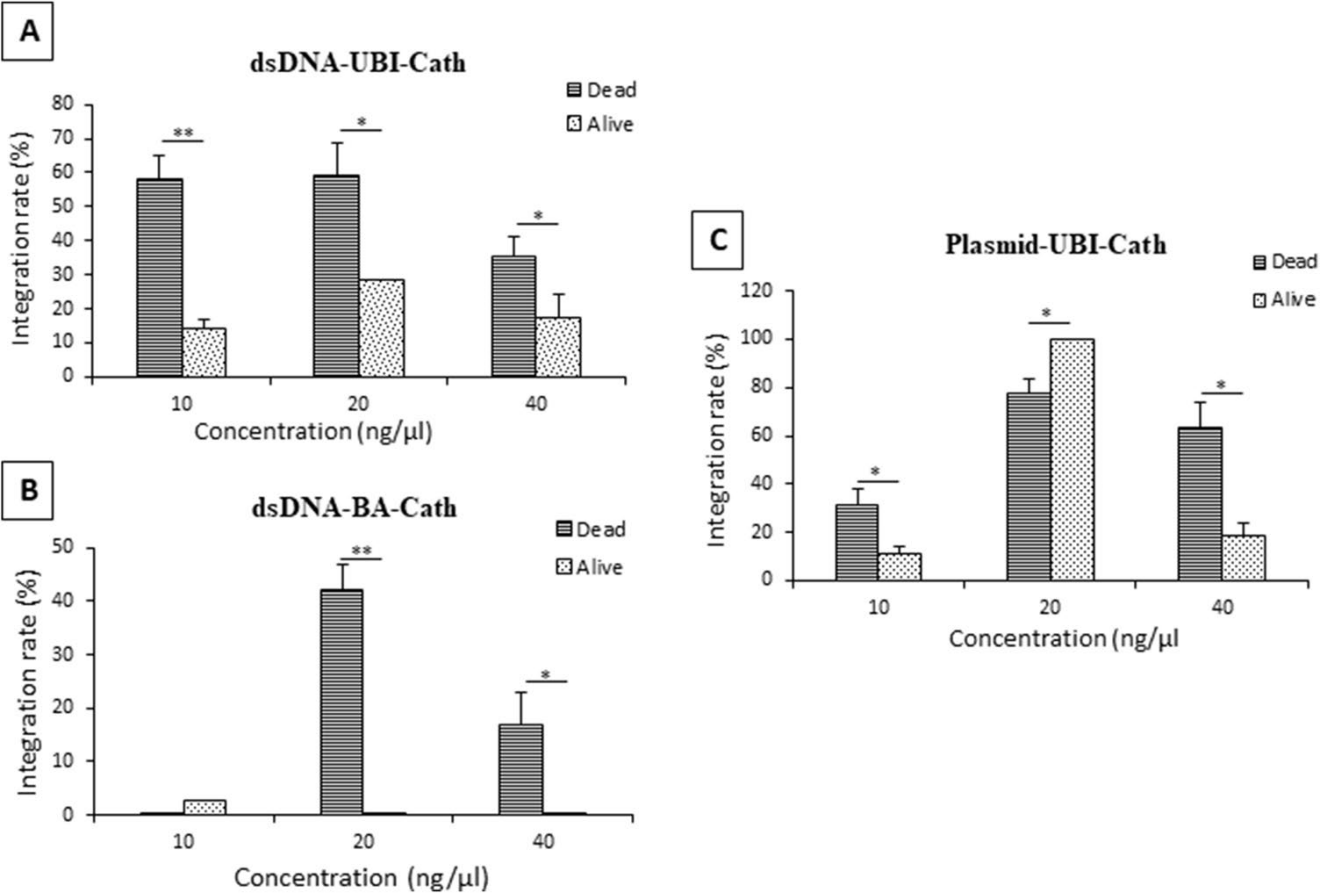
Comparison of integration rates between dead and alive fish of different transgene constructs carrying alligator cathelicidin gene in channel catfish (*Ictalurus punctatus*) using CRISPR/Cas9 knock-in system. (**A**) dsDNA construct driven by zebrafish ubiquitin promoter (dsDNA-UBI-Cath) (*N* for dead fry: 49, 43 and 23 for 10, 20 and 40 ng/µL, respectively; *N* for alive fingerlings: 73, 9 and 88 for 10, 20 and 40 ng/µL, respectively) (**B**) dsDNA construct driven by carp β-actin promoter (dsDNA-BA-Cath) (*N* for dead fry: 8, 37 and 35 for 10, 20 and 40 ng/µL, respectively; *N* for alive fingerlings: 118, 25 and 27 for 10, 20 and 40 ng/µL, respectively) (**C**) plasmid DNA construct with zebrafish ubiquitin promoter (plasmid-UBI-Cath) (*N* for dead fry: 42, 16 and 18 for 10, 20 and 40 ng/µL, respectively; *N* for alive fingerlings: 51, 4 and 19 for 10, 20 and 40 ng/µL, respectively). The values represent mean ± SD and paired *t*-tests were used to compare integration rates between dead and alive fish positive for transgene (*p < 0.05, **p < 0.01).

Overall, dead fry had higher integration rates compared to living fry (Fig. 2). Among living fry, the dsDNA-BA-Cath construct had much lower integration rates with only 2.6% in the lowest dosage treatment and no integration for living fry at higher dosages (Fig. 2B). High integration rates in dead fry were found for 20 and 40 ng/μL concentrations of plasmid-UBI-Cath construct at 78% and 64%, respectively (*p* < 0.05) (Fig. 2C). No integration was observed for 10 ng/μL concentrations of dsDNA-BA-Cath construct. For fingerlings, highest integration rate was found for the 20 ng/μL concentration of dsDNA-UBI-Cath construct (29%) (*p* < 0.05) (Fig. 2A), excluding the 100% integration rate found in 40 ng/μL concentration of plasmid-UBI-Cath construct, which had only two surviving fish which are positive for the transgene. The lowest integration rate was found for the 10 ng/μL concentration of dsDNA-BA-Cath construct (3%) (*p* < 0.05), while no positive fish were found for the 20 and 40 ng/μL concentrations of dsDNA-BA-Cath construct.

Pearson’s correlation test revealed a negative correlation between dosage and integration rate in dead fry for dsDNA-UBI-Cath and dsDNA-BA-Cath constructs (*p* = 0.005). As the dosage is increased for these two constructs, the integration rate decreases. There was an observed positive correlation between the dosage and integration rate in dead fry for plasmid-UBI-Cath construct, but it was not significant (*p* = 0.167). As indicated above, there was no linear relationship for integration and dose for the UBI constructs as the medium concentration, 20 ng/μL gave the highest integration rates for live fry.

**Figure 3:**
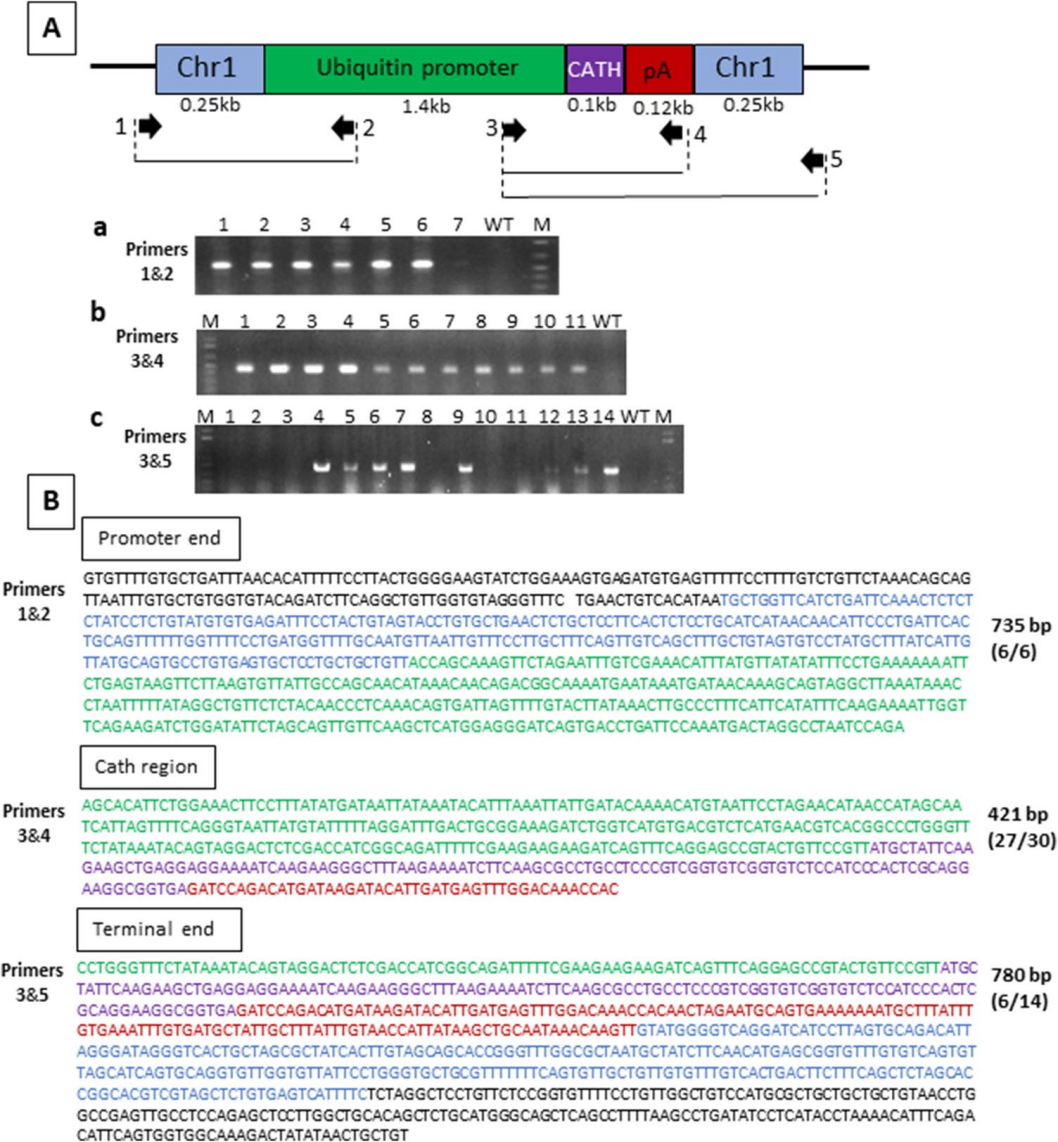
Genotyping strategy for CRISPR/Cas9 knock-in of channel catfish (*Ictalurus punctatus*) using dsDNA construct driven by zebrafish ubiquitin promoter carrying the alligator cathelicidin gene (dsDNA-UBI-Cath). (**A**) Schematic diagram of dsDNA-UBI-Cath construct. (a, c) PCR amplification of primer sets at the 5′ and 3′ junctional regions (primer sets 1&2 and 3&5) and (b) the insert-specific region for alligator cathelicidin gene (primer set 3&4). Numbers in a lane represent individual samples of fish, lane WT represent wild type channel catfish and lane M indicates DNA marker. Presence of a distinct band indicates positive for transgene. Gel electrophoresis images shown here are cropped; full-length gels are presented in Supplementary Figure S1. (**B**) Representative sequences derived from channel catfish positive for integration of the dsDNA-UBI-Cath construct. Sequences in black are the regions outside of homologous arms; Blue sequences are the homologous arms that are part of the transgene construct; Green are partial sequences from the ubiquitin promoter region; Purple are the sequences of alligator cathelicidin gene. Red sequences belong to the poly A terminator sequence. Numbers on the right side of each sequence indicate the number of base pairs in the promoter region, alligator cathelicidin gene region and terminal region as revealed by sequencing of positive fish. Numbers in parentheses are the number of sequencing reactions which yielded positive transgene integration over the total number of sequencing reactions.

**Figure 4:**
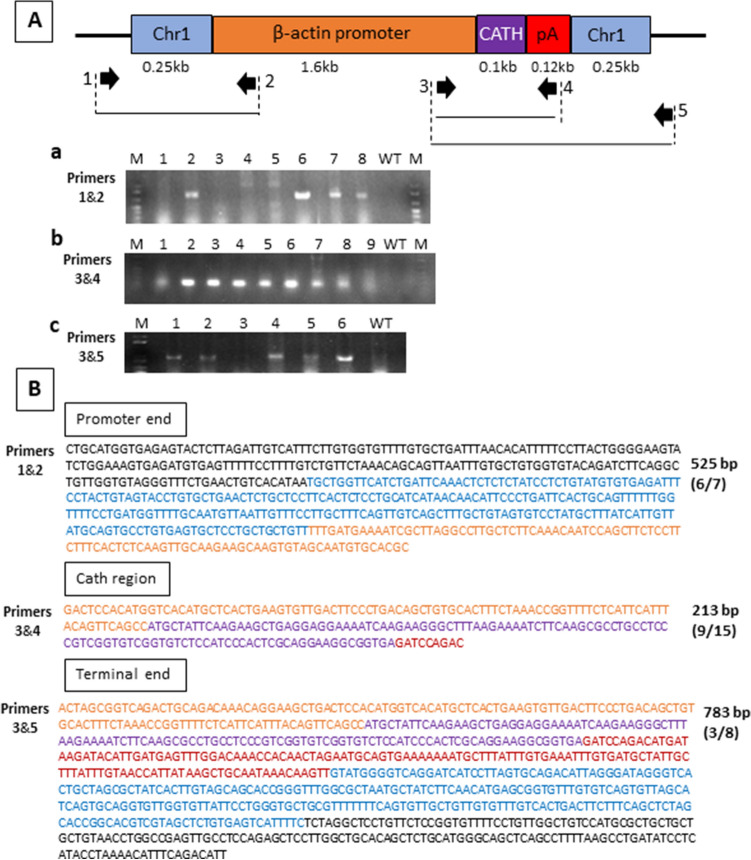
Genotyping strategy for CRISPR/Cas9 knock-in of channel catfish (*Ictalurus punctatus*) using dsDNA construct driven by β-actin promoter carrying the alligator cathelicidin gene (dsDNA-BA-Cath). (**A**) Schematic diagram of dsDNA-BA-Cath construct. (a, c) PCR amplification of primer sets at the 5′ and 3′ junctional regions (primer sets 1&2 and 3&5) and (b) the insert-specific region for alligator cathelicidin gene (primer set 3&4). Numbers in a lane represent individual samples of fish, lane WT represent wild type channel catfish and lane M indicates DNA marker. Presence of a distinct band indicates positive for transgene. Gel electrophoresis images shown here are cropped; full-length gels are presented in Supplementary Figure S2. (**B**) Representative sequences derived from channel catfish positive for integration of dsDNA-BA-Cath construct. Sequences in black are the regions outside of homologous arms; Blue sequences are the homologous arms that are part of the transgene construct; Green are partial sequences from the zebrafish ubiquitin promoter region; Purple are the sequences of the alligator cathelicidin gene. Red sequences belong to the poly A terminator sequence. Numbers on the right side of each sequence indicate the number of base pairs in the promoter region, alligator cathelicidin gene region and terminal region as revealed by sequencing of positive fish. Numbers in parentheses are the number of sequencing reactions which yielded positive transgene integration over the total number of sequencing reactions.

Distinct bands amplified the different regions of the dsDNA-UBI-Cath and dsDNA-BA-Cath transgene constructs (Figs. 3A and 4A, respectively). The presence of sequences that were not part of the transgene construct (designated by sequences in black, Figs. 3B and 4B), but matched the sequences surrounding the homology arms in chromosome 1 of channel catfish further confirmed integration of the whole transgene construct.

### Microinjection decreases embryo hatchability

**Figure 5:**
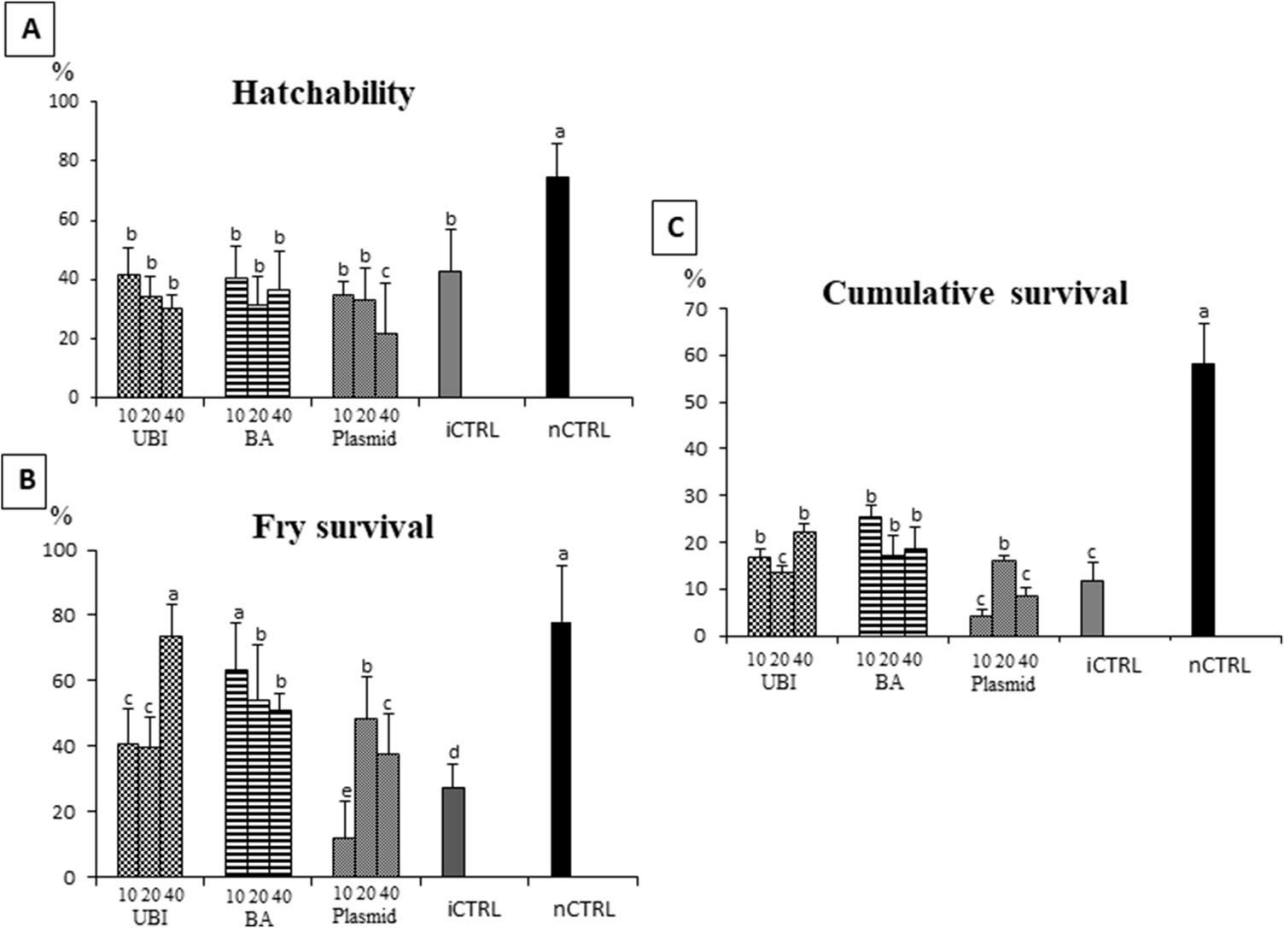
Plots of (**A**) embryo hatchability, (**B**) early fry survival and (**C**) cumulative mortality (hatchability x early fry survival) of channel catfish (*Ictalurus punctatus*) microinjected at one-cell stage with three transgene constructs: UBI (dsDNA driven by zebrafish ubiquitin promoter), BA (dsDNA driven by carp β-actin promoter) and plasmid (plasmid DNA construct with zebrafish ubiquitin promoter) at different concentrations (10, 20 and 40 ng/μL) carrying the alligator cathelicidin gene utilizing the CRISPR/Cas9 system. Control groups included the injected control (iCTRL, 60% phenol red solution only) and non-injected control (nCTRL). The values represent mean ± SD and analyzed by one-way ANOVA followed by Tukey’s test. Means with different letters are significantly different (*p* < 0.05); *N* = 300–435 embryos injected.

Hatch rate of embryos ranged from 75% in the non-injected control group to 22% in the 40 ng/μL concentration of plasmid-UBI-Cath construct (Fig. 5A). No significant differences were detected in the hatchability of microinjected embryos among the different concentrations of donor DNA for both dsDNA donor treatments. However, for the plasmid-UBI-Cath treatment, a low hatch rate was obtained at the 40 ng/μL concentration as compared to 10 and 20 ng/μL concentrations (*p* < 0.05). The non-injected control group had the highest hatch rate when compared to all other groups (*p* < 0.01). No significant correlation was detected among the different concentrations of donor DNA and embryo hatchability for dsDNA-BA-Cath construct, but a negative correlation was found to be significant (*p* = 0.019) between dosage and hatchability for the plasmid-UBI-Cath construct and dsDNA-UBI-Cath (*p* = 0.058). In general, the embryo hatchability decreased as the donor DNA concentration increased for these two constructs.

### Microinjection harms early fry survival

Significant differences in fry survival rates were detected among the different concentrations of each donor DNA construct (*p* < 0.05) (Fig. 5B). For dsDNA-UBI-Cath construct, the 40 ng/μL concentration showed the highest fry survival rate (73%) as compared to two other dosages. In addition, a positive correlation (*p* = 0.001) was also found between dosage and fry survival for the different concentrations of dsDNA-UBI-Cath construct: the higher the dosage of donor DNA used, the higher the fry survival. For dsDNA-BA-Cath construct, high fry survival rate (63%) was observed in 10 ng/μL concentration. However, no correlation was found between dosage and fry survival (*p* = 0.186). In contrast, the 10 ng/μL concentration of plasmid-UBI-Cath construct gave the lowest fry survival rate (12%) as compared to other concentrations, but no correlation was found between dosage and fry survival (*p* = 0.163). The non-injected control group had the highest fry survival (78%) as compared to all other treatment groups (*p* < 0.01) and their controls. The injected control group had the lowest fry survival as compared to all other treatment groups (*p* < 0.01).

For the cumulative survival, the highest rate was found in the non-injected control group (58%) and the lowest in the 10 ng/μL concentration of plasmid-UBI-Cath construct (4%) (Fig. 5C). A positive correlation (*p* = 0.030) was found between dosage and cumulative survival for the different concentrations of dsDNA-UBI-Cath construct. However, no correlation was found between dosage and cumulative survival for dsDNA-BA-Cath construct (*p* = 0.210) and plasmid-UBI-Cath construct (*p* = 0.627). In general, the plasmid-UBI-Cath construct treatments had low cumulative survival similar to the injected control.

### Transgene expression detected in most tissues of P_1_ fish

**Figure 6:**
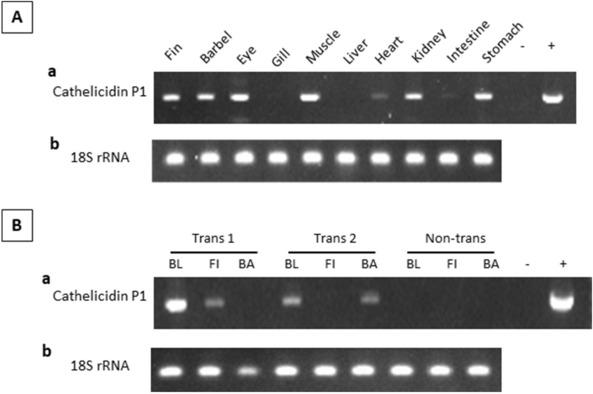
Expression of alligator cathelicidin mRNA from the dsDNA-zebrafish ubiquitin-alligator cathelicidin construct in (**A**) different tissues and (**B**) tissues biopsied non-lethally from P_1_ transgenic channel catfish (*Ictalurus punctatus*), as measured by regular RT-PCR followed by agarose gel electrophoresis. Trans 1 and Trans 2 were P_1_ transgenic fish and Non-trans was a non-transgenic fish. *BL* blood, *FI* fin, *BA* barbel; + , plasmid DNA positive control; -, PCR reaction in the absence of plasmid DNA. 18S rRNA gene was used as an internal control. Gel electrophoresis images were cropped and full-length gels are presented in Supplementary Figure S3.

To detect the expression of cathelicidin transgene in P_1_ fish (parent generation; wild type fish injected with Cas9, sgRNA and a transgene construct), total RNA samples isolated from various tissues of positive fish were subjected to RT-PCR and qPCR analyses. Figure 6Aa shows that 8 out 10 tissues expressed the cathelicidin transgene. Strong expression was observed in fin, barbel, eye, muscle, kidney and stomach, while weak expression was observed in heart and intestine. No expression was detected in gill and liver. To avoid sacrificing potential transgenic fish, blood, fin and barbel were biopsied to isolate RNA. Representative results of cathelicidin transgene expression in two randomly selected positive fish as compared to a non-transgenic fish (analyzed negative for cathelicidin transgene) are shown in Fig. 6Ba. Transgene mRNAs were consistently detected in the blood of both transgenic fish, but the expression in the fin and barbel varied. Gel images of the 18S rRNA used as internal control are shown in Fig. 6Ab and Bb.

**Figure 7:**
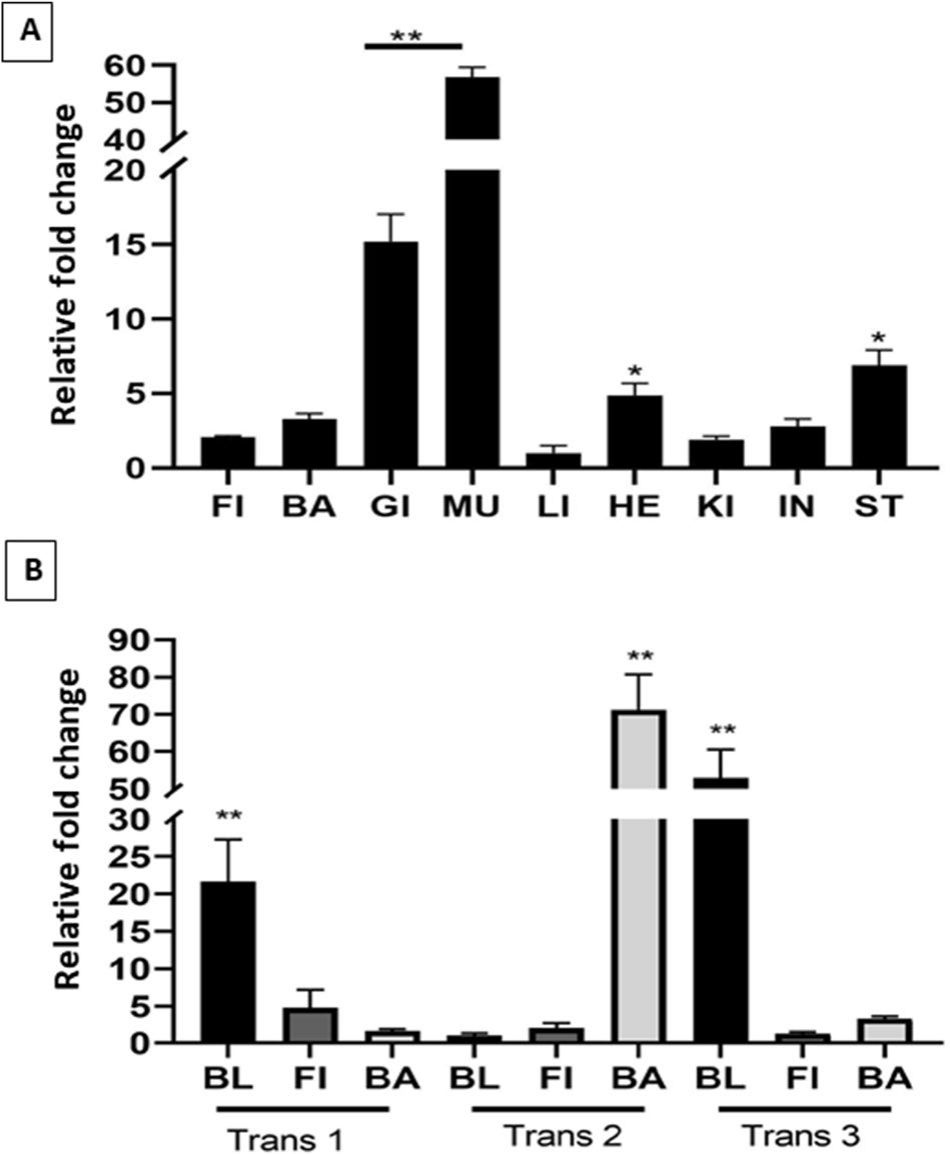
Relative expression profiles of the dsDNA-zebrafish ubiquitin-alligator cathelicidin construct in (**A**) different tissues and (**B**) tissues biopsied non-lethally from P_1_ transgenic channel catfish (*Ictalurus punctatus*), as measured by qPCR. *FI* fin, *BA* barbel, *GI* gill, *MU* muscle, *LI* liver, *HE* heart, *KI* kidney, *IN* intestine, *ST* stomach, *BL* blood. Expression levels in (**A**) were calibrated against liver tissue which had the lowest expression level, and 18S rRNA gene was used as a reference gene. Trans 1, Trans 2, and Trans 3 were P_1_ transgenic fish. Expression levels in (**B**) were calibrated against corresponding tissues from sibling non-transgenic control fish. The expression level was analyzed by 2^−ΔΔCT^ method and one-way ANOVA followed by Tukey’s test (**p < 0.001, *p < 0.01) Representative gel image in (**A**) can be visualized in Supplementary Figure S4.

To quantify the level of cathelicidin gene expression in the tissues of transgenic fish, qPCR was performed. High variation was observed among different tissues with a maximum fold change approaching 60 (Fig. 7A). Based on the fold changes relative to the liver (set as 1×), cathelicidin mRNA was most abundant in muscle by 57-fold (*p* < 0.001), followed by gill (15-fold), stomach (7-fold) and heart (5-fold). The rest of the tissues had less than threefold changes. For the biopsied tissues, three positive fish for cathelicidin gene were sampled for blood, fin and barbel. Based on the fold changes relative to the negative fish (non-transgenic), the highest fold change was found in the barbel of transgenic fish 2 (Trans 2) with a 71-fold change and in the blood of transgenic fish 3 (Trans 3), a 53-fold change (*p* < 0.001) (Fig. 7B). Relatively high fold change (*p* < 0.01) was also observed in the blood of transgenic fish 1 (Trans 1), while the rest of the tissues had less than fivefold change.

## Discussion

To facilitate a targeted knock-in approach in channel catfish using CRISPR/Cas9 system, a multi-pronged approach was undertaken. An effective donor DNA construct was designed to increase integration efficiency, and a non-coding region of the channel catfish genome was edited with CRISPR/Cas9. The target region had no gene coding regions, microRNA loci, long non-coding RNA regions or heterochromatin to hypothetically not knockout any important loci or function, to increase the probability of transgene expression and reduce potential off-target and pleiotropic effects. Then a disease-resistance alligator cathelicidin transgene was inserted into this region of the channel catfish genome. Relatively, high integration rates were achieved. This is the first report of targeted gene insertion in the non-coding region away from genes, microRNA loci, long non-coding RNA regions and heterochromatin for an aquaculture species, and as best we can tell for any organism. Several tissues of the transgenic individuals strongly expressed alligator cathelicidin.

The insertion efficiency of dsDNA donor templates in CRISPR/Cas9 system is generally poor^35^ as compared to single-stranded DNA (ssDNA) donors^36,37^. We successfully generated knock-ins using dsDNA donor templates with an integration rate as high as 59% in fry that died and 14.1–28.6% in surviving fingerlings. The results were comparable to the precise insertion rate of 60–90% using ssDNA donor templates in human cell lines^38,39^ and mouse models^37,40^. Insertion via homologous recombination of dsDNA donor template in mouse models had a knock-in efficiency of ~ 10% or less^41–43^. A knock-in efficiency of 3.5% was observed in zebrafish using a dsDNA donor oligonucleotide targeting *C13H9orf72* genomic locus but only 1.7% showed correct knock-in without additional mutations^44^. In the current study, sequence results revealed the precise integration of the complete cathelicidin gene without errors.

High integration rates ranging from 31 to 78% in dead fry and 11 to 100% in fingerlings were observed with the use of a plasmid donor template. These results were slightly better than the integration rates (via HDR) obtained using plasmid donors in medaka^30^ and zebrafish^45^ embryos, which ranged from 25 to 27% and 26 to 46%, respectively. Preliminary CRISPR/Cas9 knock-in experiment in our lab using a plasmid donor yielded a 10% integration rate for Elovl2 transgene at the same target site in channel catfish chromosome 1 (Xing, et al. unpublished results). Plasmids as donor vectors for CRISPR/Cas9 transfections are common, but their drawbacks include random integration of all or part of the plasmid DNA into the host genome, unwanted insertions of plasmid DNA sequences at on-target and off-target sites^46^. However, sequencing results obtained in this study on samples positive for the transgene with the use of plasmid donor DNA revealed that no unwanted plasmid sequences were integrated into the target site.

The efficiency of HDR generating successful knock-ins in the current study can be due to several factors. First, the design of the homologous donor construct contributed to the efficiency of HDR which led to high integration rates. Studies have shown that the efficiency of recombinatorial repair increases as the length of homology arms increases^14,46,47^. The use of 250-bp homology arms identical to the sequences surrounding the target site in channel catfish chromosome 1 generated integration rates ranging from 14 to 100% in P_1_ fingerlings. A similar finding was observed by Zhang et al.^47^ in which increased length of homology arms from 50 to 300 bp led to targeting the insertion site more effectively in 293 T cells. In that study, Zhang et al.^47^ observed a 77% precise insertion rate of 300 bp homology arms; in contrast, inserts containing 50 bp homology arms had only a 63% insertion rate. Studies that used 1 kb or more homology arms achieved variable results and showed 12–58% HDR rate^46,48^. Shorter homology arms would be expected to be more accessible for insertion in the target site during the simultaneous cleavage of genomic and plasmid DNA^48^. Also, the donor templates used were designed to have two homology arms identical to sequences surrounding DSB created by Cas9/sgRNA which allowed HDR to occur^49^, thus resulted in the correct insertion of the transgene to the target site.

The different concentrations of dsDNA and plasmid constructs affected the hatchability and mortality of the microinjected embryos. The greater the concentration of DNA injected, the lower the hatch rate, except for the 20 ng/μL concentration for the dsDNA-BA-Cath construct. The increased embryo mortality could be due to the production of toxic products at higher concentrations of donor DNA which may have adverse effects on the cells or the excess abundance of DNA itself is lethal. In general, the cumulative survival of the plasmid-UBI-Cath treatment was the lowest. This construct was slightly larger than the other two constructs consistent with the premise that increasing amounts of injected exogenous DNA would lower survival. Additionally, we did not track the rate of the degradation of the plasmid carrying the transgene into the embryo, thus another possibility is an extended period with excess exogenous DNA or other toxic effects such as cGMP-AMP synthase activation^50,51^ from the short-term persistence of plasmid sequences.

Even though they hatched successfully, the injected control fry experienced the heaviest mortality of all treatments, suggesting long-lasting negative effects of the damage of microinjection of the yolk. These comparisons suggest that major mortality occurs due to the microinjection of the yolk, and additional less impactful mortality occurs from the DNA and reagents. This is also important as it indicates that the comparison of microinjected transgenic, gene edited or xenogenic fish controls at early ages that are not microinjected would be invalid due to the long-lasting negative effects of the microinjection on growth and survival. Comparisons must be made with injected controls or in the next generation. Eight of nine DNA-injected treatments had higher survival than the injected control, suggesting that there are early positive disease resistance benefits from the alligator cathelicidin helping to correct for the vulnerabilities due to microinjection, which apparently lasted for several days. Although cathelicicin expression in the newly hatched fry was not evaluated in the current study, the expectation is that these fry were expressing the transgene since expression was found in all transgenic individuals when they were older and because ubiquitin is a constitutive promoter. The higher survival of the DNA-injected treatments illustrates their potential importance compared to other current disease control measures. Both the DNA-injected and buffer-injected controls had lower survival, likely caused by both physical damage as well as greater microbial attack due to the puncture of eggshell and yolk. Doxycycline was used to try to prevent any mortality from pathogens, but was not totally effective, especially in the non-transgenic control. Therapeutic chemicals are often not 100% effective and may have inconsistent protection or killing of disease organisms^52^, thus transgenic enhancement of disease resistance may be a better alternative for long-term disease protection.

Contradictorily, the integration rate in the fry that died after hatching had a higher integration rate than surviving fingerlings. Perhaps, some individuals are overexpressing cathelicidin in key tissues or cell types, leading to mortality. These observations should be clarified in the F_1_ generation. Elucidating the relationship of the expression of transgenic antimicrobial peptides and its effect on the microbiome might be an area to explore or the effects of alligator cathelicidin on the transcriptome. Similarly, almost all β-actin alligator cathelicidin hatchlings died. Again, perhaps, expression was too strong in certain cells or tissues, with negative effects on the microbiome or negative effects on the transcriptome. The choice of promoter appears critical, and in this case, zebrafish ubiquitin gave superior results in regards to producing viable transgenic fingerlings than carp β-actin.

Low levels of somatic mosaicism were observed in the tissues of positive P_1_ fish as revealed by RT-PCR and qPCR, another benefit of the CRISPR/Cas9 targeted knock in. This is in contrast to the universal mosaicism obtained with traditional microinjection that often results in the transgene only being present in 25–30% of the tissues in P_1_^53,54^. With the presence of the transgene in every tissue, phenotypic data collected on P_1_ individuals positive for transgene becomes more realistic, although not perfect as we did not determine if the mosaicism varied from tissue to tissue. Thus, tissue patterns of expression may or may not be the same when the F_1_ generation is produced. However, when our laboratory used CRISPR/Cas9 to produce knockouts without accompanying gene insertion, mosaicism was low and similar among all tissues evaluated^9^.

Additionally, the fact that the gene must be present in almost all tissues as expression was obtained in almost all tissues ensures higher transmission frequencies and rates to the F_1_ generation. Also, the low level of mosaicism has positive benefits for studying transgene expression. For biopsied tissues, transgene expression was consistently expressed in the blood, while variable expression was found among transgenic fish for fin and barbel. A donor DNA construct driven by zebrafish ubiquitin promoter revealed strong transgene expression across all tissues. Although, we cannot be certain of the exact relative expression among all tissues, the important and encouraging finding is that some level of expression was virtually universal.

Future studies should compare the transgene expression of alligator cathelicidin driven by the β-actin and the zebrafish ubiquitin promoter. Some studies in transgenic animals have shown that the β-actin promoter can maintain transgene expression to adulthood, but sometimes does not show significant activity in erythrocytes or fins, or several other cell types such as brain, retina, kidney and blood^55,56^. Factors relating to the differential expression pattern utilizing the β-actin promoter may be the potential cell-type-specific requirements controlling translation or chromatin maintenance and differential methylation^56,57^. On the other hand, Mosimann et al.^57^ demonstrated that zebrafish ubiquitin promoter could drive strong and ubiquitous expression of an enhanced green fluorescent protein (EGFP) reporter gene in zebrafish and the transgene revealed strong expression in all analyzed external and internal organs, including the retina, fin fold, and across all blood cell types from embryo to adulthood. A truncated version of this promoter was utilized in the design of dsDNA and plasmid DNA construct in the current study, which might explain the high transgene expression observed in the blood of positive fish microinjected with donor DNA constructs driven by zebrafish ubiquitin promoter. The localization of cathelicidins in the myeloid tissues might also explain why high transgene expression was found in the blood of positive P_1_ fish. Myeloid tissues arise from hematopoietic stem cells in the bone marrow and represent the major leukocytes in the peripheral blood^58^. Cathelicidins are said to be stored in neutrophils and macrophages^59^, which are types of leukocytes and both possess a nucleus which can express genes.

High expression in the blood and gill of the alligator cathelicidin gene in channel catfish, as well as some of the other tissues is encouraging, and is predictive of potential disease resistance. However, the variability in expression from one individual to another may indicate that selection will likely be needed within the transgenic population to maximize disease resistance. This is not surprising as usually transgenesis and selection are needed to maximize transgenic performance^60^. The ultimate transgenic line cannot be claimed without examining the F_1_ lines and perhaps beyond.

## Conclusion

In this study, we demonstrated that targeted gene integration can be performed using CRISPR/Cas9 knock-in system in channel catfish. Effective design of a donor template incorporating the zebrafish ubiquitin promoter, presence of a 250-bp homologous arms at each end of the construct and a sgRNA designed to cleave at the exact insertion site, all contributed to increased HDR rate, leading to precise integration of the transgene construct into chromosome 1 of channel catfish genome. Evaluation of the P_1_ individuals revealed low levels of somatic mosaicism. This can be due precise genome editing by CRISPR/Cas9 and timing of introduction of Cas9 into one-cell stage embryos. The highest integration rate occurred among live P_1_ fish treated with the dsDNA-UBI-Cath construct at 20 ng/μL. The plasmid-UBI-Cath construct can be as efficient in generating transgene containing the P_1_ fish with increased integration rates, but lower early hatch and survival. To the best of our knowledge, this study represents the first description of a targeted exogenous gene insertion in a non-coding region with no microRNA loci, long non-coding RNA regions or heterochromatin using CRISPR/Cas9 system. This led to moderate to high expression in a multitude of tissues, and in the future transgene expression at this site and others should be compared to see if targeting such regions is beneficial for gene expression and phenotypic alteration. Our findings for improving the efficiency of the CRSPR/Cas9 knock-in system may be applicable to other aquaculture species as well since it represents a highly effective and more precise method of transgene insertion than those previously used in aquaculture.

## Methods

### Ethical statement

Channel catfish were reared at the Fish Genetics Research Unit of the EW Shell Research Center, School of Fisheries, Aquaculture and Aquatic Sciences at Auburn University, Alabama, USA. All experimental protocols used in this study were approved by the Auburn University Institutional Animal Care and Use Committee (AU-IACUC). All experiments on animals were performed in accordance with the Association for Assessment and Accreditation of Laboratory Animal Care (AAALAC) protocols and guidelines.

### Identification of the target sequence for gene insertion

The targeted genomic area was to be in the non-coding region away from genes, microRNA loci, long non-coding RNA regions and heterochromatin as to avoid knocking out any important functions and to increase the probability of transgene expression. Bioinformatics was conducted to identify a genomic target site and its surrounding genes in channel catfish that met this criterion using The Ensembl genome browser^61^. The channel catfish genome^62^ (IpCoco_1.2) was used and the selection was done manually.

### Design of donor DNA and preparation of sgRNA and CRISPR/Cas9 system

The coding sequence for cathelicidin gene was derived from the mature peptide sequence of *A. mississippiensis* (AM-CATH36; GeneBank accession number AKHW00000000.3)^28^. Two different promoters were used for the double-stranded DNA (dsDNA) construct; the truncated version of the zebrafish (*Danio rerio*) ubiquitin promoter (1.4 kb) previously tested by Mosimann et al.^57^ and the common carp (*Cyprinus carpio*) β-actin promoter (1.6 kb)^63^. Expression of cathelicidin gene was driven by zebrafish ubiquitin promoter for plasmid DNA construct, pUCIDT Amp (2.7 kb). Two homology arms (250 bp each) derived from chromosome 1 of channel catfish (Database ID: NC_030416.1) spanning bases 19,128,968 to 19,129,468 were placed at the left and right ends of the donor constructs. The dsDNA constructs and the plasmid DNA were synthesized by Integrated DNA Technologies (IDT) (Coralville, Iowa, USA).

The CRISPR design online tool (CRISPR Guide RNA Design Tool, Benching, https://zlab.bio/guide-design-resources) was used to design the sgRNA that targeted the channel catfish chromosome 1. The protospacer adjacent motif (PAM) sequence (5′-TGG-3′) immediately followed the 20 bp target sequence (5′-GTGCTCCTGCTGCTGTTGTA-3′), spanning 19,129,201 to 19,129,221 bp of the chromosome 1 domain. A cloning-free (PCR-based) method was used to generate sgRNA. Table 1 shows the sequences of the universal primer and sgRNA used in this study. The sgRNAs were generated by T7 run-off^2,64^. The universal primer and ssDNA templates were annealed and filled by Platinum *Taq* DNA Polymerase (Invitrogen, Waltham, MA). The resulting dsDNA served as the template for in vitro transcription to generate sgRNA using the Maxiscript T7 Kit (Thermo Fisher Scientific, Waltham, MA) and was purified using the RNA Clean and Concentrator Kit (Zymo Research, Irvine, CA). The Cas9 protein, which served as the RNA-guided DNA endonuclease enzyme, was obtained from PNA BIO Inc. (Newbury Park, CA). Three different concentrations of the donor DNA for each of the three DNA constructs were prepared: 10, 20, and 40 ng/μL, for a total of nine sets of injection solutions per trial. The CRISPR/Cas9 system used in microinjection was composed of sgRNA, Cas9 protein and donor DNA in the ratio of 1:1:1, including one component of phenol red (60%) to visually track microinjected eggs. The final concentrations of sgRNA and Cas9 protein were 150–200 ng/μL and 300–350 ng/μL, respectively. The sgRNA and Cas9 protein mixtures were incubated in ice for 8 min prior to the addition of donor DNA and phenol red, and then the mixtures were loaded into the microinjection needle^65^. There were two control groups: the injected control (without the CRISPR/Cas9 components) (iCTRL) and the non-injected control (nCTRL).

### Egg collection, sperm preparation and fertilization

Broodstock preparation and artificial spawning were performed according to Elaswad et al.^65^ with modifications. Briefly, sexually mature channel catfish males and females were selected for artificial spawning. Female fish were implanted with 100 μg/kg of luteinizing hormone releasing hormone analog (LHRHa) to induce ovulation, and then eggs were stripped in a 20-cm greased spawning pan. Males were euthanized, their testes collected, crushed and sperm prepared in 0.9% saline solution. Sperm suspensions (1–2 mL) were added to the eggs and mixed gently. To activate the sperm, sufficient fresh water was added to the eggs to cover the mass; the sperm/egg mixture was gently swirled for 30 s. More fresh water was added and the eggs were allowed to harden for 10–15 min before microinjection.

### Microinjection and hatching of embryos

To insert the donor template carrying the cathelicidin gene, we used the small guide RNA (sgRNA) (Table 1) that directed Cas9 nuclease to that specific location in a non-coding region of chromosome 1. The designed sgRNA was co-injected with Cas9 along with each donor DNA constructs in different concentrations (10, 20, and 40 ng/μL).

The microinjection solution mentioned above was injected into one-cell stage embryos as described by Khalil et al.^2^ using a microinjection system from Applied Scientific Instrumentation (Eugene, OR). Briefly, 50 nL of the solution was directly injected into the yolk sac of each embryo using a 1.0 mm OD borosilicate glass capillary that was previously pulled into a needle by a vertical needle puller (David Kopf Instruments, Tujunga, CA). Embryos were injected within 15–90 min post-fertilization. The injected and control embryos were then reared in 10-L tubs filled with Holtfreter’s solution (59 mmol NaCl, 0.67 mmol KCl, 2.4 mmol NaHCO_3_, 0.76 mmol CaCl_2_, 1.67 mmol MgSO_4_)^66,67^ containing 10 ppm doxycycline. The embryos were incubated with continuous aeration at 27 °C for 6–8 days until hatching. Dead embryos were recorded and removed daily. Those that hatched were transferred to a Holtfreter’s solution without doxycycline until swim up. They were then fed with *Artemia* nauplii three or four times a day once their yolk sac was absorbed. Early fry survival was measured at 15 days post hatch and the live fry were reared in 60-L recirculating aquaria systems.

### Integration analysis

Genomic DNA from dead fry and fin-clipped samples of 2- to 3-month-old fingerlings was extracted via proteinase K digestion and iso-propanol precipitation as previously described^68^. Genotyping strategy had two steps: first, amplification of the cathelicidin region of the donor DNA construct to confirm insertion of the gene, and second, amplification of the 5′ and 3′ junctional regions to ensure that promoter and terminal regions were also inserted. Primers were designed using Primer3Plus software and listed in Table 2. For ease of genotyping a large number of individuals, primer pairs that could amplify the cathelicidin region for both ubiquitin and β-actin constructs were subjected to PCR amplification. Subsequently, those positive samples were further tested for PCR amplification of 5′ and 3′ junctional regions in the transgene construct, promoter and terminal ends, respectively, so as to test for proper integration on both ends of the transgene. The same primer sets were used for the dsDNA ubiquitin and plasmid DNA ubiquitin constructs. A positive band indicated a correctly oriented knock-in at the targeted locus. PCR products from individual fry were gel extracted from a 1% agarose gel and verified by Sanger sequencing performed by Genewiz (South Plainfield, NJ). Integration rates were calculated as the number of positive individuals detected by PCR in a replicate or treatment divided by the total number of individuals in the same replicate or treatment multiplied by 100.

**Table 2:** Oligonucleotide primers used in determining integration and transgene expression of alligator cathelicidin gene in channel catfish (*Ictalurus punctatus*).

Target gene	Purpose	Name	Nucleotide sequence (5′ → 3′)
Cathelicidin P1 transgene	PCR: Cathelicidin region (Ubiquitin promoter)	Ubi-Cath-F1	GCAGCCAATCACTGCTTGTA
		Ubi-Cath-R1	GTGGTTTGTCCAAACTCATCAA
	PCR: Promoter end (Ubiquitin promoter)	Ubi-PE-F2	GGCTGTTGGTGTAGGGTTTC
		Ubi-PE-R2	GCAGCTAGTGAGTGCTGTGC
	PCR:Terminal end (Ubiquitin promoter)	Ubi-Chr1-F1	GCGGAAAGATCTGGTCATGT
		Ba-Chr1-R2	CAAGTGCAAAGAAGGCAACA
	PCR:Cathelicidin region (β-actin promoter)	Ba-Cath-F1	GACTCCACATGGTCACATGC
		Ba-Cath-R1	GTCTGGATCTCACCGCCTTC
	PCR:Promoter end (β-actin promoter)	Chr1-Ba-F1	CTGTGCTGCTGATGACCATT
		Chr1-Ba-R1	GCGTGCACATTGCTACACTT
		Chr1-Ba-R2	GGCAGATGATATTCCGCACT
	PCR:Terminal end (β-actin promoter)	Ba-Cath-F1	GACTCCACATGGTCACATGC
		Ba-Chr1-R1	TGTTGCCTTCTTTGCACTTG
	qPCR	Ubi-qPCR-F1	TGCTATTCAAGAAGCTGAGGAGG
		Ubi-qPCR-R1	TCATGTCTGGATCTCACCGC
18S rRNA	qPCR	18sF	GAGAAACGGCTACCACATCC
		18sR	GATACGCTCATTCCGATTACAG

### Determination of transgene expression

Expression of the cathelicidin transgene in positive P_1_ individuals was determined by reverse transcription (RT)-PCR and quantitative real-time PCR (qPCR) analyses. RT-PCR analysis was performed as described previously^26^ with modifications. In brief, total RNA was isolated from various tissues such as fin, barbel, gill, muscle, liver, heart, kidney, intestine and stomach using RNeasy Plus Universal Mini Kit (QIAGEN), and blood samples using RiboPure-Blood Kit (Life Technologies, Carlsbad, CA). One microgram of total RNA was reverse transcribed using iScript cDNA Synthesis Kit (Bio-Rad) in a 10-μL reaction volume according to manufacturer’s protocol. Then, 1 μL of cDNA solution was subjected to PCR amplification in a volume of 10 μL containing 3.75 μL nuclease-free water, 0.25 μM of each gene specific primer and 1 μL of EconoTaq Plus 2× Master Mix (Lucigen, Middleton, WI). The PCR amplification procedure was as follows: initial denaturation for 3 min at 95 °C, followed by 35 cycles of denaturation at 95 °C for 30 s, annealing at 58 °C for 30 s and 1 min extension at 72 °C, and a final extension at 72 °C for additional 5 min. The PCR products were analyzed by electrophoresis on 1% agarose gels.

For qPCR analysis, all cDNA products from P_1_ transgenic channel catfish were diluted to 200 ng/µL and analyzed with a CFX96 real-time PCR Detection System^69^ (Bio-Rad Laboratories, Hercules, CA). Each amplification was performed in a 10-µL reaction volume containing 5 µL of SsoFast EvaGreen Supermix (Bio-Rad), 1 µL of 5 µM forward and reverse primers (Table 2), 2 µL nuclease-free water and 1 µL of cDNA. The reaction conditions were as follows: 94 °C for 5 s, followed by 40 cycles of 94 °C for 5 s, 60 °C for 5 s, and a dissociation curve profile of 65–95 °C for 5 s/0.5 °C increment. To quantify the gene expression of alligator cathelicidin driven by zebrafish ubiquitin promoter in the different tissues of P_1_ transgenic channel catfish (N = 3), expression levels of different tissues were calibrated against the liver tissue of these same fish which had the lowest expression value (arbitrarily set to 1×). To evaluate the alligator cathelicidin effect between P_1_ transgenic and sibling non-transgenic channel catfish, the biopsied tissues such as blood, fin and barbel were used and relative gene expression was calibrated against corresponding tissues from sibling non-transgenic fish using the CFX Manager Software version 1.6 (Bio-Rad), and crossing-point (C_T_) values were converted to fold differences using the relative quantification method. Each sample was performed in triplicate and every analysis was performed using the formula 2^(−ΔΔCT)^^70^ which sets the zero expression of the negative control (non-transgenic full-siblings) to 1× for comparison. To normalize mRNA expression levels, 18S rRNA was used as internal control. PCR using primers Ubi-qPCR-F1 and Ubi-qPCR-R1 should amplify a fragment of 123 bp (Table 2). Samples for qPCR were resolved on 1% agarose gel and representative gel image in Fig. 7A can also be visualized in Supplementary Figure S4.

### Statistical analysis

Hatching percentage for embryos in four independent experiments was calculated as the total number of fry that has completed hatching divided by the total number of embryos, multiplied by 100. Hatching was completed and recorded after 6- or 7-days post fertilization (dpf). Fry survival was determined as the total number of fry that survived 15 days post hatch (dph) divided by the total number of hatched embryos, multiplied by 100. Cumulative survival was calculated as the number of fry that survived divided by the total number of day 0 embryos, multiplied by 100. Integration rates for each treatment were calculated as the total number of positive fish divided by the total number of fish analyzed multiplied by 100. One-way ANOVA and Tukey’s multiple comparisons test were used to analyze these data for significant differences among treatments. Histograms were generated in Microsoft Excel 2016 and GraphPad Prism 8 (GraphPad Software, San Diego, CA). Pearson’s correlation coefficient was performed to determine the relationship between integration rates and concentration of DNA constructs used in microinjection. Paired *t*-tests were used to compare integration rates between dead and alive fish positive for transgene. The Shapiro–Wilk test was utilized for analysis of data normality. All statistical analyses were performed using R software (R Core Team, 2014). Statistical significance was set at *p* < 0.05, and all data were presented as the mean ± standard deviation (SD).

## Supplementary Information

Supplementary Figures.

## Data Availability

All data generated or analyzed during this study are included in this published article (and its Supplementary Information file).
