# Supplementary material for: CRISPR/Cas9-mediated knock-in of alligator cathelicidin gene in a non-coding region of channel catfish genome
Source: Sci Rep. 2020 Dec 17;10:22271. doi: 10.1038/s41598-020-79409-5 (PMC7746764; doi:10.1038/s41598-020-79409-5)
Supplement: Supplementary file 1 — Supplementary Figures. [file 41598_2020_79409_MOESM1_ESM.pdf]

Supplementary Information

CRISPR/Cas9-mediated Knock-in of Alligator Cathelicidin Gene in a Non-coding Region of Channel Catfish Genome

Rhoda Mae C. Simora[1,2,*], De Xing[1], Max R. Bangs[3], Wenwen Wang[1], Xiaoli Ma[1], Baofeng Su[1], Mohd G. Q. Khan[4], Zhenkui Qin[5], Cuiyu Lu[1], Veronica Alston[1], Darshika Hettiarachchi[1], Andrew Johnson[1], Shangjia Li[1], Michael Coogan[1], Jeremy Gurbatow[1], Jeffery S. Terhune[1], Xu Wang[6, 7] and Rex A. Dunham[1, *]

[1]School of Fisheries, Aquaculture and Aquatic Sciences, Auburn University, Auburn, AL, 36849, USA
[2] Current address: College of Fisheries and Ocean Sciences, University of the Philippines Visayas, Miagao, Iloilo, 5023, Philippines
[3] Current address: Department of Biological Science, Florida State University, Tallahassee, FL, 32304, USA
[4] Current address: Department of Fisheries Biology and Genetics, Bangladesh Agricultural University, Mymensingh, 2202, Bangladesh
[5] Ministry of Education Key Laboratory of Marine Genetics and Breeding, College of Marine Life Sciences, Ocean University of China, Qingdao, 266003, China
[6] Department of Pathobiology, Auburn Auburn University, Auburn, AL, 36849, USA
[7] HudsonAlpha Institute for Biotechnology, Huntsville, AL, 35806, USA

*email: rcsimora@up.edu.ph; dunhara@auburn.edu

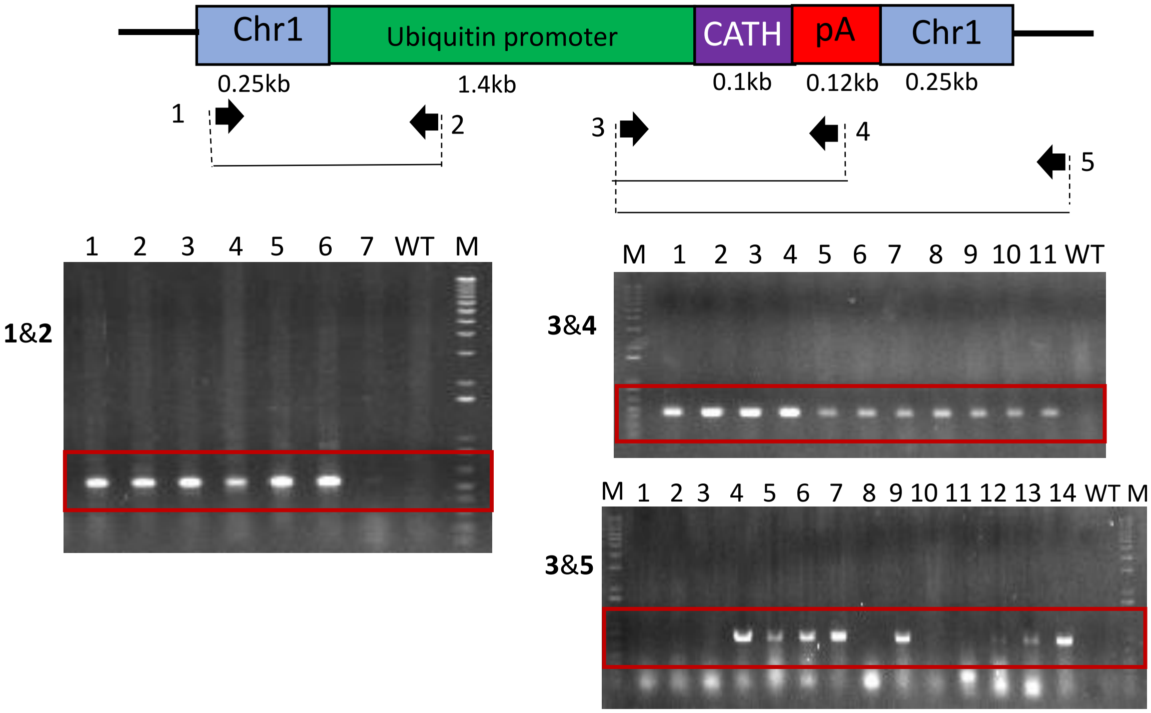

Supplementary Figure S1. Original image of gels in Fig. 4A. Red box represents the cropped area.

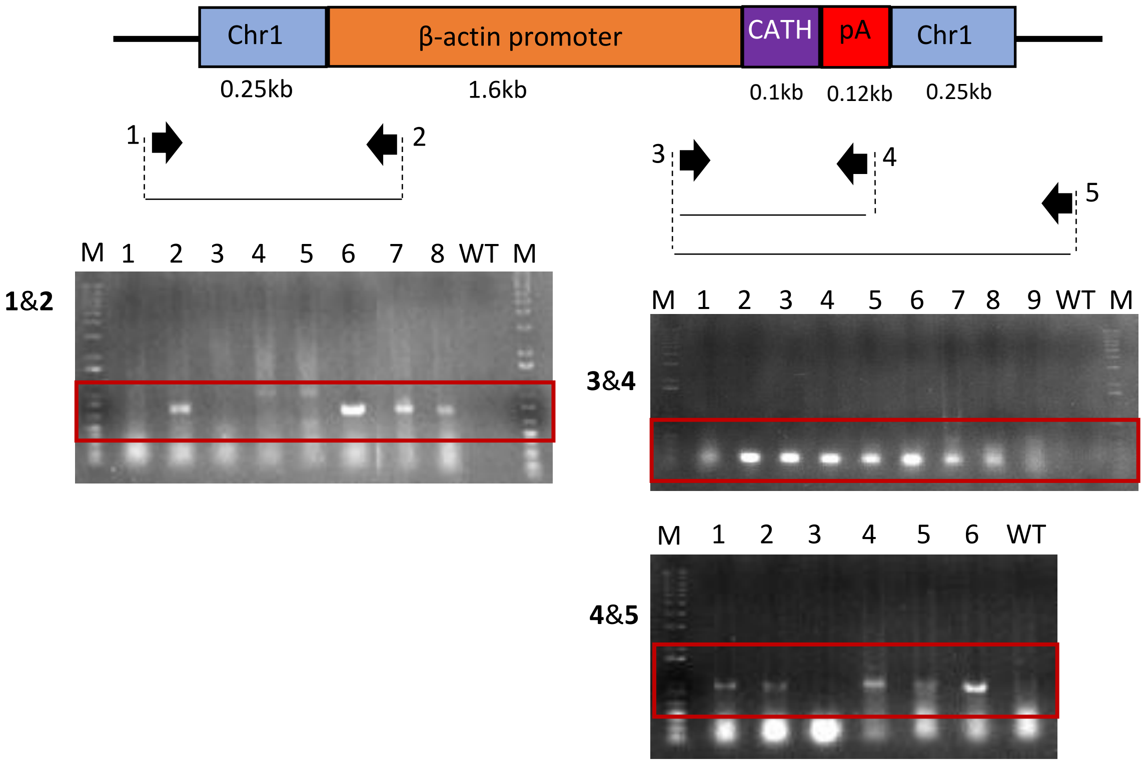

Supplementary Figure S2. Original image of gels in Fig. 5A. Red box represents the cropped area.

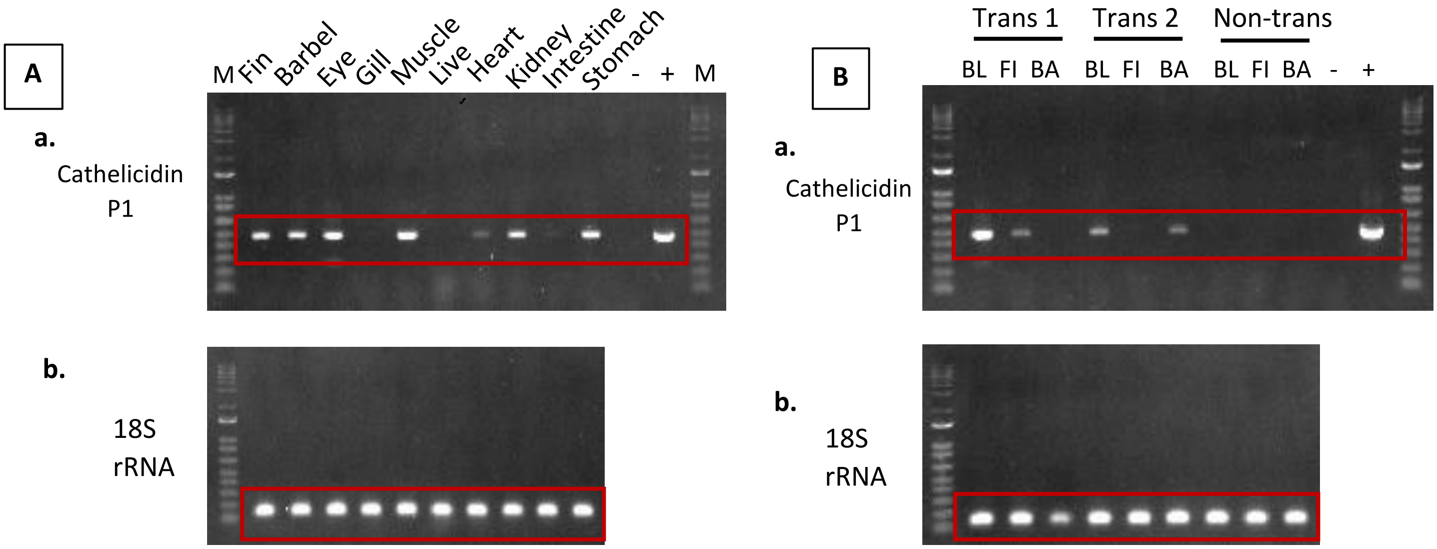

Supplementary Figure S3. Original image of gels in Fig. 6 (A, B). Red box represents the cropped area.

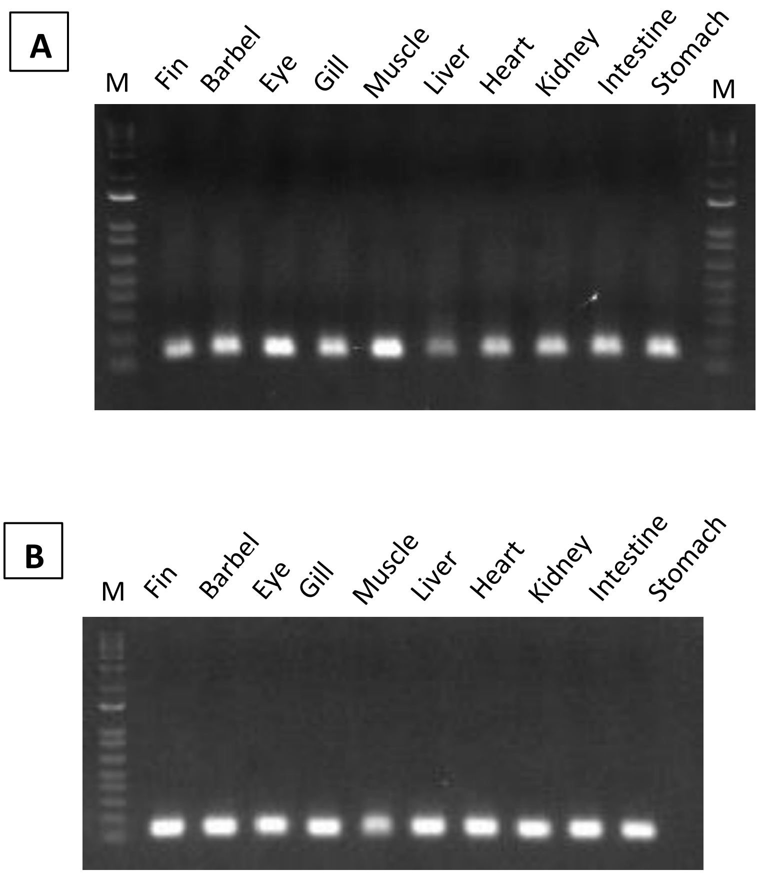

Supplementary Figure S4. Representative gel image of qPCR results for (**A**) different tissues from P_1 transgenic channel catfish (*Ictalurus punctatus*) and (**B**) 18S rRNA gene used as a reference gene in Figure 7A.
